# Long-term surgical outcome and impact on daily life activities of strabismus surgery in thyroid-associated ophthalmopathy with and without previous orbital decompression

**DOI:** 10.1186/s13005-024-00423-3

**Published:** 2024-04-01

**Authors:** Matilde Roda, Nicola Valsecchi, Natalie di Geronimo, Andrea Repaci, Valentina Vicennati, Uberto Pagotto, Michela Fresina, Luigi Fontana, Costantino Schiavi

**Affiliations:** 1 Ophthalmology Unit, DIMEC, University of Bologna, Via Pelagio Palagi 9, 4038 Bologna, Italy; 2 IRCCS Azienda Ospedaliero-Universitaria di Bologna, Bologna, Italy; 3 Endocrinology Unit, Department of Medical and Surgical Sciences, S. Orsola-Malpighi Hospital, Alma Mater Studiorum-University of Bologna, Bologna, Italy

**Keywords:** Thyroid Associated Ophthalmopathy, Strabismus, Orbital decompression, Surgical outcome

## Abstract

**Backgrounds:**

To report the long-term surgical outcomes and the impact on daily life activities of strabismus surgery in patients with Thyroid Associated Orbitopathy (TAO) with and without previous orbital decompression.

**Methods:**

Patients who underwent strabismus surgery for TAO were retrospectively reviewed. The primary outcome was to evaluate the influence of orbital decompression on the outcomes of TAO related strabismus surgery. Surgical success was defined by the resolution of diplopia and a post-operative deviation < 10 prism diopters (PD). The secondary outcomes were the clinical features, surgical approaches, and impact on daily life activities.

**Results:**

A total of 45 patients were included in the study. The decompression surgery group (DS) included 21 patients (46.7%), whereas the non-decompression surgery group (NDS) patients were 24 (53.3%). The mean follow-up time from the last strabismus surgery was 2,8 years (range 8–200 months). Successful surgical outcome was achieved in 57,1% of patients in the DS, and 75% of patients in the NDS (*p* = 0,226). DS patients required almost twice the number of surgical interventions for strabismus compared to the NDS (1,95 vs. 1,16 respectively, *p* = 0,006), a higher number of extraocular muscles recessed in the first surgery (2,67 vs. 1,08 respectively, *p* < 0.001), and a lower rate of unidirectional surgery compared to NDS (23% vs. 95%, *p* < 0,001). At the pre-operative assessment, 71.4% of DS patients had eso-hypotropia, while no patients had this type of strabismus in the NDS group (*p* < 0.001). On the other hand, the hypotropia rate was 79.2% in NDS patients and only 4.8% in DS patients (*p* < 0.001). Moreover, 21,8% of NDS patients used prism lenses in daily life activities, compared to 42.9% of patients that used prism lenses to reduce the impairment in their daily life activities (*p* = 0.016).

**Conclusions:**

The results of our study showed that DS patients required almost twice the number of strabismus surgical procedures, a higher number of extraocular muscles recessed in the first surgery, and an increased need for prism lenses to correct the residual deviation compared to the NDS, but with similar long-term surgical outcomes.

## Introduction

Thyroid-associated ophthalmopathy (TAO) is an inflammatory orbital disease of autoimmune origin that can cause severe functional and psychosocial effects. It represents one of the most difficult challenges in the clinical practice of ophthalmology [1]. It is commonly associated with hyperthyroidism, especially Graves’disease, but it can also be associated with primary hypothyroidism, Hashimoto’s thyroiditis, and sometimes in euthyroid individuals [2, 3]. TAO is relatively rare, with an estimated prevalence of 16 and 1,2 cases per 100.000 people in women and men respectively in the United States, and between 0.1% and 0.3% in the European population [4].

TAO presents two sight-threatening complications: *corneal exposure keratopathy*, due to the proptosis, and *optic neuropathy*, due to the stretching of the optic nerve secondary to the proptosis and to the compression of the optic nerve by congested tissues and extraocular muscles. In these circumstances, orbital decompression is the only effective treatment to prevent irreversible vision loss, consisting of bony wall and fat removal [5]. Various surgical techniques have been proposed, including lateral decompression, inferomedial or antral-ethmoidal decompression (translid, transcaruncular and transantral approach), ‘three-wall’ decompression (lateral + inferomedial approach, or coronal approach) and ‘four-wall’ decompression [6]. 

TAO-related strabismus may occur in 15% of all patients, resulting in diplopia that interferes with daily life activities [7]. Strabismus could be caused by muscle edema and inflammatory cells infiltration of the extraocular during the inflammatory phase of TAO, or by muscle fibrotic changes, occurring during the inactive phase [8]. Moreover, orbital decompression surgery may cause onset of a new strabismus or worsening of a pre-existing diplopia [9]. Clinical pictures are highly variable, but they mostly show alterations of ocular motility toward a restrictive pattern.

Several previous studies have compared the outcome of strabismus surgery on patients with and without previous orbital decompression. However, the exact role of orbital decompression is unclear in the outcomes of strabismus surgery [10]. 

The present study aims to describe the clinical features, surgical outcomes, and impact on daily life activities in TAO patients after strabismus surgery with and without previous orbital decompression.

## Materials and methods

### Study population

The medical records of patients who underwent surgery for TAO-related strabismus at Sant’Orsola-Malpighi Hospital in Bologna (Italy) from January 2012 to December 2020 were retrospectively reviewed. The study was conducted by the principles of the Declaration of Helsinki. It was approved by the local ethics committee of the local health service of Bologna, Italy (Cod CE: 432/2022/Oss/AOUBo). All patients gave informed consent.

This stud primary goal was to evaluate if surgical orbital decompression influenced surgical outcomes of thyroid-eye disease-related strabismus. Secondary goals were evaluation of the clinical features of TAO-related strabismus, surgical approaches, and the impact of the surgical outcomes on daily life activities.

Inclusion criteria were TAO-related strabismus; clinical activity score (CAS) < 3 at time of squint surgery; a time lag of at least six months after orbital decompression; no history of ocular trauma; no history of previous strabismus surgery; no history of amblyopia; no history of ocular, neurologic or systemic diseases which can interfere with ocular alignment; any known cause of interruption of binocular fusion mechanisms.

Clinical variable investigated were age, gender, age at diplopia onset, age at the diagnosis of TAO, age at orbital decompression, age at strabismus surgery, neuroimaging, pre-operative angle of deviation, type of deviation, surgical procedures, the persistence of diplopia after surgery.

All patients were operated under general anesthesia by two surgeons (CS and MF). Patients were divided into two subgroups: those who previously underwent an orbital decompression, defined as the decompression surgery group (DS), and those who did not, defined as the non-decompression surgery group (NDS). Surgical success was defined by the resolution of diplopia and a post-operative deviation < 10 prism diopters (PD) measured at two consecutive ophthalmological evaluations, as previously described [11]. 

The impact of strabismus surgery (ISS) on daily activities was evaluated through phone calls performed between April and May 2022, by asking the patient if their ocular conditions interfered with their daily life activities. We included 4 sub-groups as described. See Table 1.

**Table 1:** The impact of strabismus surgery (ISS) on daily activities in TAO patients

	Is your ocular condition interfering with your daily life activities (driving, reading, walking, working)?
ISS 1	No impairment
ISS 2	No impairment when using prismatic lenses
ISS 3	Mild impairment: no limitations of autonomy in daily activities using prismatic lenses
ISS 4	Severe impairment: limitation of autonomy in daily activities using prismatic lenses

### Statistical analysis

For statistical analysis, normality was tested with the Shapiro–Wilk test and parametric tests were used. The Chi-square test was used to measure the association between two categorical variables. T-Test was used to compare variables between decompressed and non-decompressed TAO patients. *P* values < 0.05 were considered statistically significant. Statistical analysis was performed using IBM Statistical Package for Social Sciences (SPSS), version 26.

## Results

### Demographic characteristics and pre-operative assessment

The study included 45 patients, 16 men (35.6%) and 29 women (64.4%), with a mean age of 55 ± 10 years. The DS group included 21 patients (46.7%), whereas the NDS patients were 24 (53.3%).

Thirty-seven patients presented a diagnosis of Graves’ Disease (82,2%), four patients presented a diagnosis of Hashimoto (8,9%), and four patients were euthyroid (8,9%).

Orbital decompression was performed in cases when visual function was threatened by corneal exposure or optic nerve compression. None of the patients underwent decompression exclusively for cosmetic reasons. Seventeen patients were treated with inferomedial decompression through a trans-antral approach (80.9%), 1 patient was treated with inferomedial decompression through a trans-lid approach (4.7%), 2 patients underwent three-wall coronal decompression (9.5%), and 1 patient lateral decompression (4,7%). The primary indication for strabismus surgery in all patients was to relieve diplopia. Diplopia was present before orbital decompression in 47% of patients, whereas 53% of patients complained of a new onset of diplopia after orbital decompression.

At the pre-operative assessment, 15 DS patients (71.4%) had eso-hypotropia, while no patients had this type of strabismus in the NDS group (*p* < 0.001). Among the 15 DS patients with eso-hypotropia, 14 cases underwent inferomedial decompression through a trans-antral approach, whereas 1 case underwent lateral decompression. On the other hand, the hypotropia rate was 79.2% in NDS patients and only 4.8% in DS patients (*p* < 0.001). The patient with hypotropia in the DS group underwent inferomedial decompression through a trans-lid approach. Five patients had only esotropia before surgery in DS (23,8%), compared to two patients in the NDS (8,3%). Among the 5 DS patients with esotropia, 3 underwent inferomedial decompression through a trans-antral approach, and 2 underwent three-wall coronal decompression. All patients presented restrictive pattern strabismus. Before the first strabismus operation, the patients in the DS had esotropia that averaged 37,9 +/- 18 prism-diopters (PD) compared with 19.5 PD +/- 9 in the NDS (*p* = 0,175). The average amount of hypotropia was 17,9 +/- 7,7 PD in the DS and 24,9 PD +/- 7,8 in the NDS (*p* = 0,025). See Table 2.

**Table 2:** Demographic and surgical features between decompression surgery (DS) and non-decompression surgery groups (NDS)

	Decompression group	Non-decompression group	***P*** value
Number of patients (%)	21 (46,7%)	24 (53,3%)	
Age, mean (SD)	50 ± 9	57 ± 9	0,053
Females, number (%)	14 (66.7%)	15 (62.5%)	0,371
Grave’s disease	19	18	
Hashimoto’s thyroiditis	2	2	
Euthyroidism	0	4	
Pre-surgical Eso-Hypotropia	15 (71,4%)	0	< 0.001
Pre-surgical Esotropia	5 (23,8%)	2 (8,3%)	
Pre-surgical Hypotropia	1 (4,8%)	19 (79,2%)	< 0.001
Pre-operative Esotropia (PD) mean +/- SD	37,9 +/- 18	19,5 +/- 9	0,175
Pre-operative Hypotropia (PD) mean +/- SD	17,9 +/- 7,7	24,9 +/- 7,8	0,025

### Surgical intervention

The mean time between orbital decompression and strabismus surgery was 1 year ± 3 months.

The first surgical procedure was the weakening of the restricted muscles, whereas strengthening procedures were used in subsequent surgeries.

Only 5 of 21 patients in the DS had unidirectional surgery (i.e., inferior or medial rectus muscle recessions only), whereas 23 of 24 patients in the NDS had unidirectional surgery (*p* < 0,001).

Overall, the reoperation rate was 35.6%. In the DS, 9 required only one surgical intervention (42,9%), 5 patients required 2 surgeries, 6 patients required 3 surgeries and 1 patient required 4 surgeries, with a mean of 1,95 surgeries per patient. On the other hand, in the NDS, only 4 patients required 2 surgeries, with a mean of 1,16 surgeries per patient (*p* = 0,006). Comparing the two groups, patients who underwent more than one surgical procedure for strabismus correction were 57,1% in the DS, compared to 16,7% in NDS (*p* = 0.006). Moreover, the mean number of extraocular muscles recessed in the first surgery was 1,08 in the NDS, compared to 2,67 in the DS (*p* < 0.001).

### Post-operative assessment

The mean follow-up time from the last strabismus surgery and the most recent follow-up visit was 2,8 years (range 8 months – 200 months). Overall, the surgical success rate (defined by the resolution of diplopia and a post-operative deviation < 10 PD measured at two consecutive ophthalmological evaluations) was 70%. In the DS, surgical success was achieved in 57,1% of patients, whereas NDS showed a rate of residual strabismus of 75% (*p* = 0,226). When post-operative diplopia was present, the prescription of prism lenses was sufficient to correct the diplopia in 60% of patients. In the DS, patients with diplopia before orbital decompression did not present a different outcome compared to patients who presented diplopia after decompression (*p* = 0,653).

### The impact of strabismus on daily life activities

After a mean of 6,1 years (range 4,6–273 months) from the last strabismus surgery, 62,2% of patients did not refer significant impairment in daily life activities, 22.2% did not have significant impairment when using prismatic lenses, 6.7% reported mild impairment in the daily life activities while using prism lenses, and 8.9% expressed severe impairment with limitation of autonomy in the daily life activities even with prism lenses.

Seventeen of 21 patients in the DS presented no limitations in daily life activities, compared to 22 of 24 patients in the NDS (*p* = 0,292). Moreover, 21,8% of of NDS patients used prism lenses in daily life activities, compared to 42.9% of DS patients that used prism lenses in order to reduce the impairment in their daily life activities (*p* = 0.016). In the DS, those patients who complained of diplopia before orbital decompression did not present a different rate of impairment in daily life activities compared to patients who presented strabismus after decompression (*p* = 0,361).

## Discussion

Few studies discussed and compared presentation and management of strabismus surgery in TAO patients with and without orbital decompression [12–15]. 

The main finding of our study was that DS with TAO showed a lower but comparable surgical outcome to NDS (success in 57,1% vs. 75% respectively, *p* = 0,226) after a mean follow-up of 2,8 years. In the literature, the outcome of strabismus surgery in TAO patients is highly variable, with a success rate ranging from 47 to 86%. Perhaps, DS patients required almost twice the number of surgical interventions for strabismus compared to the NDS (1,95 vs. 1,16 respectively, *p* = 0,006), a higher number of extraocular muscles recessed in the first surgery (2,67 vs. 1,08 respectively, *p* < 0.001), and a lower rate of unidirectional surgery compared to NDS (23% vs. 95%, *p* < 0,001).

A previous study by Ruttum found that patients who had undergone prior orbital decompression for compressive neuropathy or corneal exposure had a lower success rate compared to the NDS, with a higher number of muscles operated after an average follow-up of 16 months, and a lower rate of unidirectional surgery required. However, in that study, the number of strabismus surgeries required was similar for the two groups (1,4 vs. 1,2) [13].

A recent study by Lee et al. compared the outcomes of strabismus surgery in patients with TAO who had undergone bone removal orbital decompression (BROD) and fat removal orbital decompression (FROD) with those who had not undergone any orbital decompression in patients with at least six months follow-up. In this study, they reported a higher number of muscles operated, and a higher number of surgeries required in the decompression group compared to NDS (1,6 vs. 1,2), but no statistically significant difference between the three groups in terms of surgical outcomes [14]. 

On the other hand, Gilbert et al. reported no differences in surgical outcomes between DS patients treated for the risk of neuropathy or corneal exposure and NDS patients. Also, they found that DS patients required a higher number of muscles operated on compared to NDS, but no differences in terms of the number of surgeries (1,2 vs. 1,2) and the rate of unidirectional surgery between the two groups [12]. Moreover, Kim et al. found no differences in terms of surgical outcomes, average number of operated muscles, and need for reintervention between DS and NDS patients after a mean follow-up of 25 months (2,4 vs. 2,2). However, in their study, orbital decompression was mainly indicated for cosmetic reasons [15]. 

The results of our study suggest that the complexity of strabismus in TAO patients is greater in those that had a previous orbital decompression than in those without, but with comparable long-term surgical outcomes. This difference observed between the two groups may be explained by the decompression surgery itself and the underlying ophthalmopathy. Orbital decompression may be responsible for the formation of scars and adhesions around the extraocular muscles, resulting in subsequent strabismus surgeries being more challenging with less predictable outcomes. Also, as suggested by Ruttum, the need for orbital decompression in patients with TAO reflects the worse degree of ophthalmopathy, negatively affecting strabismus surgery outcomes [13]. On the other hand, when patients were treated with decompression for cosmetic reasons, there were no differences in terms of surgical outcomes, suggesting that ophthalmopathy was less severe in these patients.

Another result of our study was the difference in terms of preoperative type of strabismus between NDS and DS groups (*p* < 0.001). NDS patients presented vertical or horizontal strabismus, especially a hypotropia or an esotropia. This result reflected the frequency of extraocular muscles involvement in TAO: the inferior rectus muscle is the most common muscle affected, followed by the medial rectus muscle [1]. Conversely, DS patients presented a restrictive eso-hypotropia, which may result from varied and combined mechanisms, such as localized soft tissue swelling, soft tissue and muscle entrapment, or mechanical restriction secondary to damage peribulbar tissues and orbital bones [16]. 

The orbital decompression is likely to subvert the orbital anatomy, and it could justify the higher number of muscles operated, and the higher number of surgeries required in the DS compared to NDS. In the present study, the most frequent decompression surgery performed was inferomedial decompression through a trans antral approach, accounting for 80.9% of cases. While praised for its efficacy and lack of visible scarring, this approach is also known to elevate post-operative motility imbalance by restricting abduction and/or elevation [17]. Therefore, the results of our study confirm the highest incidence of induced diplopia after the trans antral approach, but also suggest that strabismus could be present after any type of decompression technique. Future studies stratifying patients according to the different decompression surgical techniques are needed in order to understand how different approaches can impact long-term outcomes after strabismus surgery.

Thus, the pathogenesis of TAO-related strabismus differs between DS and NDS groups, requiring different surgical approaches. In the NDS, unidirectional and one-muscle surgery led to good surgical outcomes, and in most cases, one surgery was enough to resolve the diplopia and ameliorate the ocular motility. In the DS, it was necessary to perform bidirectional surgery and multi-muscle surgery, with a higher number of surgical procedures.

Regarding the impact on daily activities, 62,2% of patients did not have any impairment after a mean of 6 years from surgery, suggesting that successful surgical outcomes significantly improved quality of life in TAO patients. Nonetheless, the DS most frequently used prism lenses in daily life activity in order to relieve the diplopia compared to NDS (57.1% vs. 20.8%, *p* = 0.016). Prism lenses are effective in correcting the residual strabismus after strabismus surgery, but their use is associated with side effects, such as headaches, dizziness, eye strain, and pulling [18, 19]. 

The main strength of our study is the long-term follow-up. To the best of our knowledge, this is the study with the longest follow-up assessing the effects of orbital decompression on strabismus surgery in patients with TAO. Limitations of our study are the retrospective nature, and the relatively limited number of patients. Furthermore, additional investigations are necessary to evaluate the outcomes of strabismus surgery in patients who undergo decompression for cosmetic purposes compared to those who receive surgery in cases where visual function is at risk due to corneal exposure or optic nerve compression.

## Conclusion

The management of TAO-related strabismus is challenging, especially in TAO patients with previous orbital decompression. Our study showed that DS patients required almost twice the number of strabismus surgical procedures, a higher number of extraocular muscles recessed in the first surgery, and an increased need for prism lenses in order to correct the residual deviation compared to the NDS, but with similar long-term surgical outcomes. Thereupon, patients who are offered orbital decompression should be informed that potential subsequent strabismus would require more surgical procedures than non-decompressed patients, with an increased need for prism lenses in order to correct the residual deviation.

## Data Availability

The datasets generated during the current study are not publicly available due to protection of the patient?s personal information but are available from the corresponding author on request.

## Abbreviations

TAO: Thyroid-associated ophthalmopathy
CAS: clinical activity score
DS: decompression surgery
NDS: non decompression surgery
PD: prism diopters
ISS: impact of strabismus surgery on daily activities
BROD: bone removal orbital decompression
FROD: fat removal orbital decompression
